# Promoting Interprofessional Education and Collaborative Practice in Rural Health Settings: Learnings from a State-Wide Multi-Methods Study

**DOI:** 10.3390/ijerph18105162

**Published:** 2021-05-13

**Authors:** Priya Martin, Alison Pighills, Vanessa Burge, Geoff Argus, Lynne Sinclair

**Affiliations:** 1 Cunningham Centre, Darling Downs Health, Toowoomba, QLD 4350, Australia; 2 Faculty of Medicine, Rural Clinical School, The University of Queensland, Toowoomba, QLD 4350, Australia; 3 Mackay Institute of Research and Innovation, Mackay Base Hospital, Mackay, QLD 4740, Australia; Alison.Pighills@health.qld.gov.au; 4 Division of Allied Health, Darling Downs Health, Toowoomba, QLD 4350, Australia; Vanessa.Burge@health.qld.gov.au; 5 Southern Queensland Rural Health, The University of Queensland, Toowoomba, QLD 4350, Australia; G.Argus@uq.edu.au; 6 School of Psychology and Counselling, University of Southern Queensland, Toowoomba, QLD 4350, Australia; 7 Centre for Interprofessional Education, University of Toronto, Toronto, ON M5G 2C1, Canada; Lynne.Sinclair@uhn.ca

**Keywords:** interprofessional education, collaborative practice, rural healthcare teams, rural health, continuing professional development

## Abstract

Evidence is mounting regarding the positive effects of Interprofessional Education and Collaborative Practice (IPECP) on healthcare outcomes. Despite this, IPECP is only in its infancy in several Australian rural healthcare settings. Whilst some rural healthcare teams have successfully adopted an interprofessional model of service delivery, information is scarce on the factors that have enabled or hindered such a transition. Using a combination of team surveys and individual semi-structured team member interviews, data were collected on the enablers of and barriers to IPECP implementation in rural health settings in one Australian state. Using thematic analysis, three themes were developed from the interview data: IPECP remains a black box; drivers at the system level; and the power of an individual to make or break IPECP. Several recommendations have been provided to inform teams transitioning from multi-disciplinary to interprofessional models of service delivery.

## 1. Introduction

Interprofessional collaborative practice (IPCP) occurs when multiple health workers from different professional backgrounds work together with patients, families, carers and communities to deliver the highest quality of care [1]. Effective IPCP has been shown to strengthen health systems and improve health outcomes, and specifically to have positive effects on patient safety, chronic disease management, prescribing practices, healthcare costs, health service utilisation and falls reduction [1,2,3].

Health workers require specific knowledge and skills to work effectively in interprofessional teams, and interprofessional education (IPE) is an effective means to equip health workers for IPCP [4]. IPE is defined as occasions where, “two or more professions learn with, from and about each other to improve collaboration and the quality of care” [5]. A collaborative practice-ready health worker is someone who has learned how to competently work in an interprofessional team where there is collective identification of client goals through co-operation and joint intervention between the various clinicians, the clients and their family [1].

A growing number of studies have identified a range of factors that enable or constrain interprofessional education and collaborative practice (IPECP) in health settings. These include factors relating to individual health workers, health professions, teams and system-related factors. Individual factors identified as contributors to effective IPECP include curiosity and willingness to learn, humility to learn from others, openness to innovation and exposure to interprofessional learning opportunities [6,7]. Professional socialisation has been shown to have a positive or negative effect on attitudes to IPECP, depending on the profession involved [7,8], while repeated opportunities for effective, reciprocal communication or exposure to other professions through co-location was found to have a positive influence on IPECP [6,9]. Team factors that support IPECP include a shared understanding of purpose, direction and teamwork, and clarity regarding responsibilities, and understanding of one another’s roles in context [10,11]. System-related factors include supportive management systems and regulations, and a combination of top down and bottom up support [9,12].

From the limited literature specifically examining IPECP in rural health settings, some enablers and barriers to IPECP have been identified. IPECP enablers include IPECP leaders/champions, broader scope of practice, effective teamwork, role clarity, mutual respect, regular opportunities for cross-disciplinary communication and opportunities for interprofessional learning [13,14]. Barriers to IPECP in rural areas include service models, structures that result in health workers working in silos, geographic dispersal of team members, disruption of teamwork and communication, excessive workload and fatigue, individual personalities and gaps in organisational readiness for IPECP [13,14,15].

Whilst there is limited information available on what factors facilitate or hinder IPECP in rural health settings, in-depth information is needed to understand the individual, team and system-related layers in these unique contexts. As cultural factors can affect IPCP, it is necessary to understand what factors enable or hinder IPECP in particular contexts [16]. This information will enable healthcare workers and organisations to better embed IPECP in their health service delivery and can inform other healthcare teams in setting up or transitioning to an IPCP model of care. The study reported in this paper was undertaken to explore the enablers of and barriers to IPECP implementation in rural health settings in Queensland, where IPECP is still in its infancy.

## 2. Materials and Methods

### 2.1. Research Design

This multi-methods study utilised a combination of a team survey and individual team member interviews. The interview component of the study was designed using a constructivist lens, where participants and the interviewers co-constructed meaning [17]. Individual, semi-structured interviews were used to collect data.

### 2.2. Setting

This study was conducted in public health settings in four regional and rural Queensland health services.

### 2.3. Participants and Sampling

Participants from a range of health professions and experience levels including new graduates, experienced workers and team leaders/managers, were targeted from medicine, nursing and allied health. Purposive sampling was used to identify teams that used an IPCP model of care or were in the process of transitioning to an IPCP model of care.

### 2.4. Procedure

Five teams providing sub-acute, rehab, community and health promotion services were recruited. The team leader from each team completed the Assessment of Interprofessional Team Collaboration Scale (AITCS-II) for practitioners [18]. This was done to gauge the level of collaborative practice that occurred at each team, given some teams were already using an IPCP model of care and others were in the process of transitioning to one. Once teams were chosen, a purposive maximum variation sampling was used to recruit five participants within each team to represent a diverse range of professional backgrounds and experience in their profession and role. Individual, semi-structured interviews were then conducted by the first author and a research assistant, both qualitatively trained researchers, with a health professional background. Given the participants were dispersed across the state, all interviews were conducted via telephone.

### 2.5. Data Collection

The AITCS-II for practitioners was completed by the team leader from each team to determine the perceived level of collaboration in their teams, to enable meaningful interpretation of the interview findings. This instrument is based on the concept of collaborative team-based practice and has recently undergone further psychometric refinement [18]. The AITCS-II consists of 23 items that provide an overall collaboration score and three subscale scores for partnership, cooperation and coordination. Each of the items is rated on a 5-point Likert scale from 1 (never) to 5 (always). The AITCS-II has shown good internal consistency with an overall Cronbach’s α of 0.894 and for the subscales, 0.898, 0.924, 0.898 and 0.894 for partnership, cooperation, coordination and collaboration respectively. Mean scores for each domain can be categorised into three areas of team collaboration: need to focus on developing collaborative practice (1.0–2.9); moving towards collaboration (3.0–3.9); good collaboration (4.0 or more) [18].

An interview guide was developed to guide the interviews (see Appendix A). The guide consisted of a comprehensive set of open-ended questions with associated prompts. All interviews were audio-recorded with consent and transcribed by an independent typist. Every third interviewee was sent a summary of their interview for data checking to enhance the rigour of the study.

### 2.6. Analysis

Data from the AITCS-II were analysed by calculating the means and standard deviations for each subscale and the total score.

Interview data were subject to thematic analysis, a method for systematically identifying, organizing and offering insights into patterns of meaning or themes across a data set. Doing so enabled the researchers to make sense of the collective or shared meanings and experiences [19]. Data were coded by two authors (PM and AP), who employed an inductive thematic analysis approach, being driven by what was in the data. Both authors, first coded five interviews independently and met to develop a coding framework. Subsequently, both the authors met several times to refine and organise codes into categories and agree on and develop themes. Crucial to this process was the authors’ shared understanding of IPECP terminologies and concepts.

### 2.7. Ethics Approval

Ethics approval for the study was obtained from the Darling Downs Human Research Ethics committee for multi-sites (Ref: HREC/18/QTDD/38). Subsequently, site specific approvals were obtained from all the health services involved in the study.

## 3. Results

Five team leaders provided AITCS-II data. Team responses indicated that teams 4 and 5 reported good collaboration across all domains. All five teams reported good collaboration in the partnership domain with teams 1, 2 and 3 reporting moving towards collaboration in the domains of cooperation, coordination and overall collaboration. Data from the AITCS-II are presented in Table 1.

Overall, 25 individual interviews were completed, yielding nearly 14 h of audio-recorded data. Interviewees occupied junior and senior level positions and were from the following professions: dietetics, exercise physiology, nursing, occupational therapy, pharmacy, physiotherapy, podiatry, psychology, speech pathology, social work, rehab assistance and administration. Interviewees worked in a range of roles including clinical, management, administration and health promotion. Participants’ experience in their profession ranged from 2 years to over 30 years. At the time of the interview, participants had been in their current roles for between 4.5 months and 20 years. Further participant characteristics are presented in Table 2.

Thematic analysis of the interview data resulted in the development of three themes: IPECP remains a black box; drivers at the system level; and the power of an individual to make or break IPECP.

### 3.1. IPECP Remains a Black Box

It was clear that IPECP was still a mystery to many healthcare workers. This was largely due to the lack of understanding about the concept of IPECP itself, its terminology, and the confusion in differentiating an interprofessional model of care from multidisciplinary and transdisciplinary models. Participants said:

*I think interprofessional is a definition a lot of people don’t understand well. Multidisciplinary is in that traditional language…we think we’re multidisciplinary, but I feel we are just working in our little silos…*

(Int 27)

*Yes, I mean there’s still a lot to learn about this whole interprofessional collaborative practice but at this stage we’ve just been thrown together and it’s kind of worked.*

(Int 11)

Participants highlighted a need for shared language and consistent labelling of IPECP concepts and terminology. One participant talked about a lack of awareness of IPECP even within teams that engaged with components of it:

*I know interprofessional practice has been around for a while…but I’m not sure how well understood (it is) within our health service in particular…over the last couple of years or so I think there are probably lots of things that teams are actually doing that you could say that’s a component of interprofessional practice but I guess the understanding of that may not be there either.*

(Int 7)

Also raised was the need to clarify expectations at a team level and have explicit conversations about the model of care the team used, who they are and what they do as a team, plus some IPE competencies:

*I think in an ideal world it would be great before they even started doing anything if they had attended a workshop or at least had a discussion as a team about what they think interprofessional collaborative practice is. So, everyone is very clear about the team, how the team and everyone’s role will function. I think that clarity is really, really key and I think have discussions on things like conflict resolution and collaborative leadership. So that would be an ideal world.*

(Int 6)

Those who had participated in targeted IPECP professional development opportunities appeared to have a good understanding of the concept, terminology and associated resources. They were able to describe what IPECP meant and how the interprofessional model of care differed from other models such as multidisciplinary and transdisciplinary ones. One participant from a team that was involved previously in implementing an interprofessional model of student placement, and hence had received training in IPECP concepts, said:

*(I am aware of) the Canadian Interprofessional Health Collaborative. We did a few reading booklets and some diagrams on how the model works; the interprofessional education guidelines and the University of Toronto facilitating interprofessional clinical learning…there were also some resources from one of the universities in Sydney.*

(Int 17)

### 3.2. Drivers at the System Level

For IPECP uptake, implementation and sustainability, several pre-requisites were identified as important at the system level—including organisation and team levels. The drivers identified were strong leadership, structures and processes that facilitate or hinder collaboration and the impact of rurality.

#### 3.2.1. Leadership

A key IPECP driver identified was strong leadership. Frequently changing or poor leadership was seen as detrimental to IPECP. Whilst reflecting on the enablers to IPECP, one participant said:

*Our team leader is fantastic, she’s very supportive and very approachable…just having that strong support as a lead who is very approachable…and having that stable person is good.*

(Int 15)

Another participant, whilst reflecting on IPECP barriers in a recently held role, noted several challenges with a hierarchical team structure and dominance of bio-medical models:

*It’s the patients who luck out at the end of the day…. I loved the experiences out there and would have been extremely happy to stay out there if the management was supportive and the difficult personalities were dealt with in an appropriate way.*

(Int 20)

#### 3.2.2. Structures and Processes That Facilitate or Hinder Collaboration

IPECP can be facilitated only as well as the structures and processes allow. Teams that allow co-location of workers have noted more collaboration than others that are located in different offices:

*We have a nice large office where we all are, and we know each other quite well and you can say—hey can I just talk to you about this patient.*

(Int 27)

*And it (co-location) does help. Just yesterday we had this very difficult discharge and I was talking to the NDIS and hospitals in [xx—bigger centre] and consultants…and just the [xx] physio who is out here for three months came around after the phone call and said—hey did you know that we do this at the [xx] hospital and it might work for this patient…and it did.*

(Int 25)

This however can be a cause of concern for some as these conversations are not documented formally and are seen as counter-productive:

*The co-location can be great in terms of promoting discussion, but it can open it to too much discussion or too much noise in the workplace.*

(Int 15)

Teams that have embedded IPECP at multiple levels appear to have more collaboration at the point of care. This involved including IPECP in team meeting agendas, access to identified champions and undertaking team-level, targeted professional development. One participant described what they would like to see happen:

*I know there is stuff online. I haven’t looked at it…when you do a course you come back and put it on your bookshelf. You want to use it, but it disappears for some reason. I think our best resource would be setting time aside to discuss it once a month or bring it up at a team meeting or build it up from the team level…what do we want to do in interprofessional practice and go and find the resources for that.*

(Int 25)

Participants reported structural barriers as impeding interprofessional student placements, including scheduling/logistical issues and staff members working part-time. One participant noted:

*The tricky thing is… all the different disciplines have different lengths of time. The physio blocks are only five weeks, OT students have been here for ten weeks… the psych is six months. So, trying to get them all lined up can be tricky.*

(Int 15)

Such logistical barriers also hindered IPECP between the team members:

*I think being practical around looking at one another’s diaries and looking at how you set up your clinics, how they are going to work together if they’ve all got separate diaries… and not a lot of flexibility, it’s going to be hard to do joint sessions, joint therapy….*

(Int 12)

Multiple interviewees reported a lack of supported, quarantined time to engage in IPECP which was seen as a barrier to facilitating IPECP at the point of care. Concerns were raised that despite best intentions, the ripple effect from targeted professional development does not necessarily reach the point of care:

*I think it comes down to time. We were very keen to start working interprofessionally after that [xx] training but we haven’t really gotten together and worked out a plan…we just haven’t organised anything so far as professional development opportunities go, it gets put on the backburner, patient care comes first.*

(Int 24)

On the contrary, one participant praised their team leader for allowing protected time to work on IPECP:

*Our team leader has formally set aside time where our clinics have been closed off so we can devote and dedicate this time to developing strategies, actually looking at how we can change our practice to be more interprofessional.*

(Int 13)

#### 3.2.3. Impact of Rurality

Whilst some viewed rurality as a barrier to IPECP, most participants considered it an enabler to rural practice. Being less resourced than the bigger centres was reported as a driver for teams to work more collaboratively. Participants who described their rural teams as collaborative, said:

*We have to make the best of what we have and the best of people’s skills.*

(Int 16)

*Obviously lots of limited resources…and the requirement to work a bit more collaboratively so you can give the patients good care, still working within the scope of practice but yes I think that’s probably more of a necessity rather than enabled by anything in particular.*

(Int 7)

Another participant talked about getting to know the others in the team well because of the smaller size of the team:

*…this is one of the best working teams that I’ve been a part of…because it is a small team. You get to know what all of the disciplines are and what their specialty areas are, and you become aware of how to communicate with different people.*

(Int 26)

However, there appeared to be multiple barriers to IPECP at the team level including: sole positions, lack of backfill to cover leave arrangements and excessive time spent on outreach and travel thereby impeding presence at team meetings and interprofessional case conferences:

*…with some disciplines, there is only one of that team member. If they go on holidays, usually they don’t have any backfill. If someone’s on leave, there is no one from that discipline to go to.*

(Int 15)

*…as sole clinicians in rural or remote…we are out in different towns almost every day and we are not really in the same place at the same time…I guess not having regular meetings or everyone not being present at the same time.*

(Int 17)

Whilst these challenges were noted, prior planning and coordination were proposed as solutions:

*I think it’s just the disconnect that we sometimes have as a team…because all are on outreach… at four or five locations around the health service which makes it difficult to have them at the same place at the same time unless its coordinated.*

(Int 18)

### 3.3. The Power of an Individual to Make or Break IPECP

A healthcare worker who is pro-IPECP was described as having a certain set of traits, as well as being engaged in targeted professional development.

#### 3.3.1. Characteristics of a Pro-IPECP Healthcare Worker

A pro-IPECP worker was described as having client wellbeing as a focus, being open to work with others, having a sound understanding of the scope of their own role and that of the others, having respect for all team members and possessing a good set of skills such as approachability, open communication style, conflict resolution, getting on with others and self-awareness. One participant who noted good collaboration in the team said:

*I think we all get along quite well. We respect each other and as we respect each other’s space and when we need assistance, we all get together…being sensible, supportive and respectful of each other.*

(Int 9)

Another participant highlighted that the patient’s wellbeing is the central focus for those workers who are pro-IPECP:

*We see ourselves as a team first and our clinical step next…it’s just beautiful it works so well. There is no ego and we wrap around the patient, whatever they need, whatever angle needs to be addressed, for whatever reason, the person is just there.*

(Int 25)

Even pro-IPECP healthcare workers reported facing professional tribalism, with some professions demanding a higher hierarchical structure and biomedical models dominating the care agenda:

*I think there is a bit of a hierarchical kind of thing…you know the whole thing of that doctor/nurse hierarchical stuff…when you get to be in a certain position then you really don’t have to fill in your progress notes that well and you can use abbreviations and you can write badly and nobody pulls you up on it….which doesn’t really enhance interprofessional practice.*

(Int 1)

In some instances, IPECP was hindered owing to the colleagues a pro-IPECP healthcare worker must work with:

*I feel sometimes we are very multi-disciplinary and sometimes we are interprofessional. So, it depends on who I am working with as to about whether it’s more interprofessional or multi-disciplinary.*

(Int 12)

*Sometimes the risk of working with other people we’ve tried to do interprofessional with they don’t want to work together, or they really don’t care about what my role does, they just want to get in there and do their job.*

(Int 12)

#### 3.3.2. Targeted IPECP Professional Development and Follow-Up

Another important trait of a pro-IPECP healthcare worker was noted to be completion of, and follow-up from, targeted professional development:

*You can really tell the difference in people who have had training in that really collaborative practice versus more siloed practice and how willing they are able to share elements of care and responsibility.*

(Int 13)

Some raised that IPECP training needs to be also accompanied by training in related areas such as teamwork, personality styles and emotional intelligence:

*…important for the team to have some training on what interprofessional practice is….I guess training in team building activities to improve the team dynamics and team communication.*

(Int 26)

## 4. Discussion

Although rural healthcare settings are considered well-suited to IPECP implementation due to the generalist nature of rural practice, role flexibility and overlap, and the use of shared facilities by smaller teams [13,20], IPECP continues to remain at its infancy across many such settings. Our study conducted across regional and rural Queensland explored the enablers of and barriers to IPECP implementation in these settings, with a view to inform other services that are establishing or moving towards an IPCP model of health service delivery. Of the five teams included in the study, two teams had good collaboration and the other three teams were moving towards collaboration, as measured through the AITCS-II. Therefore, the findings are also relevant and applicable to teams that do not have a fully-established model of IPCP but in the process of transitioning towards one. We identified three themes: IPECP remains as a black box, drivers at the system level, and the power of an individual to make or break IPECP. Our findings clearly demonstrate that IPECP in rural health settings, similar to other health settings, is influenced by drivers at multiple levels in the healthcare system such as individual, team and organisational levels [6,7,9,10,11,12,21,22,23].

A key finding of this study is that IPECP in rural healthcare settings needs to be facilitated at multiple levels, using both top-down and bottom-up approaches. Team leaders and managers who are passionate about IPECP implementation can enable their team members to navigate some of the complex structural and process barriers to allow for more interprofessional collaboration. This is consistent with previous findings that highlight the importance of IPECP leaders and champions in implementing IPECP initiatives [13,14,22]. However, this may be negatively impacted by high levels of workforce turnover and vacancy rates that create leadership instability [24]. Embedding IPECP into team structures and processes (such as quarantining time for IPECP activities and keeping IPECP as a standing agenda item at team meetings) can empower healthcare workers to persist and make changes within their sphere of influence. There was consensus among participants that collaboration is easier when built into therapy schedules and team calendars right from the outset, especially for newer teams.

In response to the growing complexity of healthcare needs of the population globally, every healthcare worker has a role and responsibility in enhancing IPECP within their team. Participants in this study clearly described a pro-IPECP healthcare worker as someone with the patient as the focus of their own care, as one who understands the scope of their role and of others and who gets on well with their team. The importance of having such motivated healthcare workers is integral to driving the change management processes that are necessary when implementing IPECP initiatives [22].

This study highlighted the importance of targeted professional development a pro-IPECP healthcare worker undertakes. It was unsurprising that the effects of targeted training and professional development opportunities were not reaching the point of care. This again emphasizes the importance of embedding follow-up actions and a plan to progress IPECP at the team and organisational levels. Given the workforce challenges rural areas face [24], it may not be sufficient to rely on singular IPECP champions. Rural healthcare teams need to upskill multiple IPECP champions within their team to promote sustainability of IPECP initiatives. IPECP training rates in a previous Queensland survey study of 235 survey respondents (Martin et al., under review) was reported to be around 10%, exposing a need for further training in this area.

This study has again demonstrated the need for shared language and clarity of IPECP terminologies and concepts in line with previous findings [25], reinforcing the findings of the survey study by the authors which found that 81% of the participants lacked awareness of IPECP terminologies and concepts [26]. This was further highlighted by the variability in perceptions between members within the same team as to the model of care their team used. Establishing shared language can be considered a first step in driving IPECP initiatives from implementation to sustainability [21].

### Limitations

This study has some limitations. Firstly, data were collected at one point in time. We recommend that future studies consider the entire process of rural healthcare teams transitioning from multi-disciplinary to IPCP models of care. This will enable a deeper understanding of the change processes involved in the implementation and uptake of IPECP. Although our study participants represented twelve disciplines, some such as medicine were not represented. Whilst our study was also open to medical practitioners, we were unable to recruit them. This could be attributed to the high workload rural doctors face in their daily roles. We suggest that future studies use additional recruitment strategies to ensure representation from as many disciplines as possible.

## 5. Conclusions

As the rural healthcare context is quite unique, it is important to understand what works in that context to inform rural practice. In this study we explored factors that enable and hinder IPECP at the point of care in rural Australian healthcare settings. Our findings indicate that IPECP can be enhanced if individual, team and organisational structures and processes are conducive to collaboration. Several recommendations have resulted, including targeted IPECP training of healthcare professionals, use of shared language and investment in multiple IPECP champions in rural settings to cater to high staff turnover rates. These findings will be of use to rural healthcare teams that are transitioning from multi-disciplinary to an IPCP model of service delivery. Furthermore, our findings will play an important role in enhancing IPECP across Queensland, Australia, and may also have applicability to other rural healthcare settings internationally. Further studies are needed to understand the change processes that are in play when teams transition to an IPCP model of service delivery.

## Figures and Tables

**Table 1:** Sum of Scores: Means and Standard Deviations for AITCS-II.

Teams	Partnership			Cooperation			Coordination			Total—Collaboration		
	Raw Score	$\underline{x}$	σ	∑	$\underline{x}$	σ	∑	$\underline{x}$	σ	∑	$\underline{x}$	σ
Team 1	32	4.0	0	30	3.75	0.46	25	3.57	0.54	87	3.78	0.42
Team 2	32	4.0	0.54	31	3.88	0.35	23	3.29	0.49	86	3.74	0.54
Team 3	37	4.63	0.52	29	3.63	0.52	23	3.29	0.95	89	3.87	0.87
Team 4	33	4.12	0.35	39	4.88	0.35	30	4.29	0.49	102	4.43	0.51
Team 5	38	4.75	0.46	36	4.50	0.54	32	4.57	0.54	106	4.61	0.50

Note. Mean cutoff is 4.0. 1.0 to 2.9 = need to focus on developing collaborative practice; 3.0 to 3.9 = moving towards collaboration; 4.0 or more = good collaboration.

**Table 2:** Participant characteristics.

Participant Characteristics	N (25)
**Profession:**	
Nutrition and Dietetics	4
Physiotherapy	4
Nursing	3
Occupational therapy	3
Social work	3
Pharmacy	2
Exercise physiology	1
Podiatry	1
Psychology	1
Speech pathology	1
Rehab assistance	1
Administration	1
**Nature of role:**	
Clinical	18
Management	3
Both clinical and management	2
Other (e.g., admin, health promotion)	2

## Data Availability

Data and materials are protected by ethics but may be made available in de-identified format upon contact made to the corresponding author.

## Appendix A. Interview guide

1. Information about the interviewee’s team
  - Can you please start by describing how your team operates?
  - When a patient is referred to your team, what processes are followed to progress this referral and patient care?
  - Would you classify your team as multidisciplinary or interprofessional? *(definition of terms is in the fact sheet emailed to you)*
  - How does interaction between team members take place in relation to client care?
  - Can you give me an example of the best interprofessional/collaborative practice that you have witnessed in your team?
    ○ Probes—at rounds, case conferences, discharge planning meetings
  - What processes does your team use to resolve conflicts between members?
  - What professional development opportunities, if any, does your team have that incorporates upskilling in interprofessional collaborative practice? Give examples
    ○ team work, role clarification, conflict resolution, communication, collaborative leadership (*information on interprofessional team and practice has been provided to you in the fact sheet*)
  - What interprofessional collaborative practice resources have you/team members accessed? Do you know of any other existing interprofessional collaborative practice resources that you can access?
  - How do you/your team go about student placements in your unit?
    ○ Combined interprofessional tutorials
    ○ Shared teaching of students from different professions
    ○ Peer learning—students learning with students from different professions or programs
2. Enablers to interprofessional education and collaborative practice
  - What are some factors that have enabled you/your team to work collaboratively/in an interprofessional way?
    ○ Management/organisational factors
    ○ Training/Professional Development
3. Barriers to interprofessional education and collaborative practice
  - What are some factors that have hindered collaborative working in your team?
    ○ People factors (position, discipline, personality)
    ○ Workplace culture/organisational factors etc
  - Has working in a regional/rural health setting impacted on your/your team’s ability to work in a collaborative way? If yes, in what way
4. Recommendations
  - For another team setting up interprofessional collaborative practice, what recommendations or suggestions do you have?
  - Do you have any further comments on what we have discussed so far?
